# (±)-*N*-(3-Hydr­oxy-1,2-diphenyl­prop­yl)-4-methyl­benzene­sulfonamide

**DOI:** 10.1107/S1600536808028948

**Published:** 2008-09-24

**Authors:** Sok Teng Tong, David Barker, Ka Wai Choi, Peter D. W. Boyd, Margaret A. Brimble

**Affiliations:** a Department of Chemistry, University of Auckland, Private Bag 92019, Auckland, New Zealand

## Abstract

In the title compound, C_22_H_23_NO_3_S, the relative stereochemistry of the two stereogenic centres is *anti* with respect to the H atoms. The mol­ecular packing of the crystal shows a double-strand arrangement, consisting of one strand of (S*,S*) enanti­omers and one strand of (R*,R*) enanti­omers. Both strands lie parallel to each other along the *a* axis. Each strand is made up of dimers in which the mol­ecules are connected to each other *via* an inter­molecular O—H⋯O hydrogen bond between the hydroxyl groups and an O—H⋯π inter­action with the aromatic ring. These units are then connected to neighbouring dimers *via* N—H⋯O hydrogen bonds and C—H⋯O interactions. Intramolecular C—H⋯O interactions are also observed.

## Related literature

For a similar organocatalytic α-oxdiation of ketones, see: Engqvist *et al.* (2005 ▶). For a related structure, see: Chinnakali *et al.* (2007 ▶).
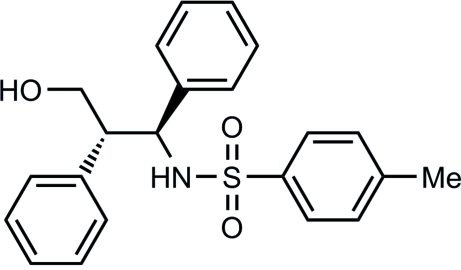


## Experimental

### 

#### Crystal data

C_22_H_23_NO_3_S
*M* *_r_* = 381.47
Monoclinic, 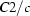

*a* = 39.4702 (16) Å
*b* = 5.4270 (2) Å
*c* = 17.4287 (7) Å
β = 91.028 (2)°
*V* = 3732.7 (3) Å^3^
*Z* = 8
Mo *K*α radiation
μ = 0.20 mm^−1^
*T* = 90 (2) K
0.4 × 0.16 × 0.14 mm

#### Data collection

Bruker SMART diffractometer with APEXII CCD detector
Absorption correction: none
22582 measured reflections
4478 independent reflections
3763 reflections with *I* > 2σ(*I*)
*R*_int_ = 0.042

#### Refinement

*R*[*F* ^2^ > 2σ(*F* ^2^)] = 0.066
*wR*(*F* ^2^) = 0.193
*S* = 1.13
4478 reflections
245 parameters
15 restraints
H-atom parameters constrained
Δρ_max_ = 1.06 e Å^−3^
Δρ_min_ = −0.64 e Å^−3^

### 

Data collection: *SMART* (Siemens, 1995 ▶); cell refinement: *SAINT* (Siemens, 1995 ▶); data reduction: *SAINT*; program(s) used to solve structure: *SHELXS97* (Sheldrick, 2008 ▶); program(s) used to refine structure: *SHELXL97* (Sheldrick, 2008 ▶); molecular graphics: *ORTEPIII* (Burnett & Johnson, 1996 ▶) and *Mercury* (Macrae *et al.*, 2006 ▶); software used to prepare material for publication: *WinGX* (Farrugia, 1999 ▶) and *publCIF* (Westrip, 2008 ▶).

## Supplementary Material

Crystal structure: contains datablocks global, I. DOI: 10.1107/S1600536808028948/pv2094sup1.cif

Structure factors: contains datablocks I. DOI: 10.1107/S1600536808028948/pv2094Isup2.hkl

Additional supplementary materials:  crystallographic information; 3D view; checkCIF report

## Figures and Tables

**Table 1:** Hydrogen-bond geometry (Å, °)

*D*—H⋯*A*	*D*—H	H⋯*A*	*D*⋯*A*	*D*—H⋯*A*
N1—H1⋯O1^i^	0.86	2.45	3.122 (3)	136
O3—H3*A*⋯O3^ii^	0.85	2.06	2.910 (5)	180
C4—H4⋯O1	0.93	2.55	2.909 (4)	104
C8—H8⋯O1	0.98	2.61	2.958 (3)	101
C1—H1*A*⋯O2^iii^	0.96	2.63	3.557 (4)	161
C1—H1*B*⋯O2^iv^	0.96	2.74	3.552 (4)	142
O3—H3*B*⋯C16^ii^	0.86	2.67	3.489 (4)	160.1
O3—H3*B*⋯C17^ii^	0.86	2.85	3.499 (4)	133.7

Symmetry codes: (i) 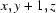; (ii) 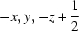; (iii) 
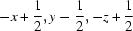
; (iv) 
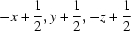
.

**Fig. 1.**
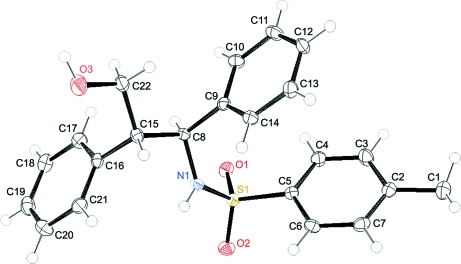
The molecular structure and atom numbering scheme of the title compound with displacement ellipsoids drawn at the 50% probability level for non-H atoms.

**Fig. 2.**
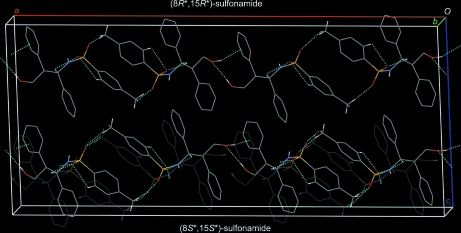
The unit cell packing of the title compound showing double strands of (8S*,15S*) and (8R*,15R*) enantiomers. A third strand of (8S*,15S*) sulfonamide (dimmed) which is positioned below the unit cell is also depicted in the figure to show the hydrogen bondings between the strands. Dashed lines represent hydrogen bonds; hydrogen atoms not involved in hydrogen bonding have been omitted for clarity.

## supplementary crystallographic information

### Comment

The title racemic sulfonamide was obtained unintentionally as a product from the study of the organocatalytic α-oxidation of phenylacetaldehyde catalysed by (*S*)-proline. The relative stereochemistry of the two stereogenic centres was established by X-ray crystallography as *anti* with respect to the H atoms of C8 and C15 (Fig. 1).

The molecular packing of the crystal shows a double strand arrangement, which consists of one strand of (8*S**,15*S**) enantiomers and one strand of (8*R**,15*R**) enantiomers. Both strands lie parallel to each other along the *a* axis and a number of hydrogen bonds has been observed throughtout the crystal lattice.

Each strand is made up of homodimeric units in which the sulfonamide molecules are connected to each other by intermolecular hydrogen bonds between the hydroxyl groups (O3—H3···O3) as well as the O—H···π interaction with the aromatic ring. The dimer is, in turn, linked to the next dimer along the strand *via* non-conventional hydrogen bonds (C1—H1A···O2—S1 and C1—H1B···O2—S1). Finally, neighbouring strand of the same stereochemistry are connected to each other *via* conventional (N1—H1···O1—S1) and non-conventional (C1—H1A···O2—S1 and C1—H1B···O2—S1) hydrogen bonds (Fig. 2).

Non-conventional intramolecular hydrogen interactions (C4—H4···O1—S1 and C8—H8···O1—S1) are also observed with a distance of 2.55 and 2.61 Å between the hydrogen and the acceptor oxygen (Table 1).

### Experimental

To a solution of 3-phenyl-2-tosyl-1,2-oxaziridine (551 mg, 2.00 mmol) in distilled THF (8 ml) was added under ambient atmosphere (*S*)-proline (69.1 mg, 0.600 mmol). After 5 minutes, phenylacetaldehyde (0.450 ml, 4.00 mmol) was added. After 1 h, sodium borohydride (151 mg, 4.00 mmol) was added to the mixture at 273 K and the mixture was stirred overnight. The mixture was then poured onto a biphasic mixture of HCl (1 mol l^-1^) and EtOAc (1:1, 8 ml) at 273 K and vigorously stirred for 10 minutes. The organic phase was separated and the aqueous phase was extracted with EtOAc (8 ml *x* 4). The combined organic extracts were washed with brine, dried over MgSO_4_ and concentrated *in vacuo* to afford a yellow oil. Purification by flash chromatography using hexane–EtOAc (2:1 to 1:1) as eluent yielded the title sulfonamide as a white solid (6%). Recrystallization of the title sulfonamide in hexane–CH_2_Cl_2_ (4:1) afforded colourless needles.

### Refinement

Hydrogen atoms attached to carbon and nitrogen atoms were placed in calculated positions and refined using the riding model (N—H = 0.86 Å & C—H 0.93–0.97 Å), with *U*_iso_(H) = 1.2 and 1.5*U*_eq_(parent atom) for the nonmethyl and methyl groups, respectively. The hydroxyl H-atom was disordered over two sites involved in either O—H···O hydrogen bonding to a neighboring alcohol or O—H···π interactions with a neighboring phenyl ring. In the final refinement these two hydrogen atoms were included, fixed in these two positions. After the final refinement a peak of electron density of 1.05 e Å^-3^, distanced 0.82 Å from the sulfonamide oxygen O2, was observed. No evidence of disorder could be discerned. This peak was also present in an alternate refinement using data that had been corrected for absorption. This refinement was indistinguishable from structure presented here.

### Crystal data

**Table:**

C_22_H_23_NO_3_S	*D*_x_ = 1.358 Mg m^−^^3^
*M**_r_* = 381.47	Melting point: 426.7(8) K
Monoclinic, *C*2/*c*	Mo *K*α radiation, λ = 0.71073 Å
*a* = 39.4702 (16) Å	Cell parameters from 6191 reflections
*b* = 5.4270 (2) Å	θ = 1.0–28.0°
*c* = 17.4287 (7) Å	µ = 0.20 mm^−^^1^
β = 91.028 (2)°	*T* = 90 K
*V* = 3732.7 (3) Å^3^	Needle, colourless
*Z* = 8	0.4 × 0.16 × 0.14 mm
*F*(000) = 1616	

### Data collection

**Table:**

Bruker SMART diffractometer with APEXII CCD detector	3763 reflections with *I* > 2σ(*I*)
Radiation source: fine-focus sealed tube	*R*_int_ = 0.042
graphite	θ_max_ = 28.0°, θ_min_ = 1.0°
ω scans	*h* = −51→51
22582 measured reflections	*k* = −7→7
4478 independent reflections	*l* = −22→22

### Refinement

**Table:**

Refinement on *F*^2^	Primary atom site location: structure-invariant direct methods
Least-squares matrix: full	Secondary atom site location: difference Fourier map
*R*[*F*^2^ > 2σ(*F*^2^)] = 0.066	Hydrogen site location: inferred from neighbouring sites
*wR*(*F*^2^) = 0.193	H-atom parameters constrained
*S* = 1.13	*w* = 1/[σ^2^(*F*_o_^2^) + (0.0794*P*)^2^ + 19.2449*P*] where *P* = (*F*_o_^2^ + 2*F*_c_^2^)/3
4478 reflections	(Δ/σ)_max_ < 0.001
245 parameters	Δρ_max_ = 1.06 e Å^−^^3^
15 restraints	Δρ_min_ = −0.64 e Å^−^^3^

### Special details

**Table:**

Geometry. All e.s.d.'s (except the e.s.d. in the dihedral angle between two l.s. planes) are estimated using the full covariance matrix. The cell e.s.d.'s are taken into account individually in the estimation of e.s.d.'s in distances, angles and torsion angles; correlations between e.s.d.'s in cell parameters are only used when they are defined by crystal symmetry. An approximate (isotropic) treatment of cell e.s.d.'s is used for estimating e.s.d.'s involving l.s. planes.
Refinement. Refinement of *F*^2^ against ALL reflections. The weighted *R*-factor *wR* and goodness of fit *S* are based on *F*^2^, conventional *R*-factors *R* are based on *F*, with *F* set to zero for negative *F*^2^. The threshold expression of *F*^2^ > σ(*F*^2^) is used only for calculating *R*-factors(gt) *etc*. and is not relevant to the choice of reflections for refinement. *R*-factors based on *F*^2^ are statistically about twice as large as those based on *F*, and *R*- factors based on ALL data will be even larger.

### Fractional atomic coordinates and isotropic or equivalent isotropic displacement parameters (Å^2^)

**Table:**

	*x*	*y*	*z*	*U*_iso_*/*U*_eq_	Occ. (<1)
S1	0.158723 (16)	0.06828 (12)	0.28375 (4)	0.01431 (19)	
O1	0.14448 (5)	−0.1745 (4)	0.27404 (12)	0.0186 (4)	
O2	0.17401 (5)	0.1376 (4)	0.35572 (11)	0.0209 (5)	
O3	0.02222 (6)	0.6152 (6)	0.18428 (13)	0.0382 (7)	
H3A	0.0092	0.6156	0.2229	0.057*	0.50
H3B	0.0008	0.5906	0.1799	0.057*	0.50
N1	0.12844 (6)	0.2624 (4)	0.26835 (13)	0.0150 (5)	
H1	0.1283	0.3959	0.2949	0.018*	
C5	0.19060 (7)	0.1017 (5)	0.21504 (15)	0.0138 (5)	
C15	0.07339 (7)	0.4164 (5)	0.23044 (15)	0.0154 (5)	
H15	0.0843	0.5788	0.2313	0.019*	
C6	0.21262 (7)	0.3020 (5)	0.22046 (16)	0.0178 (6)	
H6	0.2090	0.4253	0.2564	0.021*	
C21	0.06575 (7)	0.5353 (6)	0.36894 (17)	0.0185 (6)	
H21	0.0786	0.6753	0.3597	0.022*	
C2	0.24609 (7)	0.1333 (5)	0.11774 (16)	0.0170 (5)	
C16	0.05938 (6)	0.3698 (5)	0.30955 (15)	0.0150 (5)	
C10	0.10495 (7)	0.0475 (5)	0.07735 (16)	0.0182 (6)	
H10	0.0919	−0.0855	0.0928	0.022*	
C22	0.04555 (7)	0.4221 (6)	0.16845 (16)	0.0231 (6)	
H22A	0.0555	0.4487	0.1187	0.028*	
H22B	0.0338	0.2653	0.1672	0.028*	
C17	0.04036 (7)	0.1590 (5)	0.32496 (17)	0.0194 (6)	
H17	0.0361	0.0451	0.2861	0.023*	
C14	0.13191 (7)	0.4350 (5)	0.10406 (16)	0.0176 (6)	
H14	0.1369	0.5635	0.1377	0.021*	
C8	0.10096 (6)	0.2233 (5)	0.21110 (14)	0.0133 (5)	
H8	0.0914	0.0591	0.2196	0.016*	
C9	0.11285 (6)	0.2352 (5)	0.12954 (15)	0.0142 (5)	
C13	0.14347 (7)	0.4453 (6)	0.02985 (17)	0.0200 (6)	
H13	0.1563	0.5791	0.0141	0.024*	
C3	0.22313 (8)	−0.0595 (6)	0.11139 (18)	0.0235 (6)	
H3	0.2263	−0.1793	0.0740	0.028*	
C4	0.19557 (7)	−0.0777 (6)	0.15959 (18)	0.0213 (6)	
H4	0.1806	−0.2092	0.1547	0.026*	
C11	0.11646 (8)	0.0577 (6)	0.00254 (17)	0.0221 (6)	
H11	0.1111	−0.0685	−0.0316	0.027*	
C19	0.03415 (8)	0.2868 (6)	0.45638 (17)	0.0235 (6)	
H19	0.0257	0.2601	0.5051	0.028*	
C7	0.24002 (7)	0.3160 (6)	0.17183 (16)	0.0193 (6)	
H7	0.2546	0.4502	0.1755	0.023*	
C12	0.13588 (7)	0.2554 (6)	−0.02141 (16)	0.0205 (6)	
H12	0.1438	0.2611	−0.0713	0.025*	
C20	0.05321 (8)	0.4953 (6)	0.44183 (17)	0.0230 (6)	
H20	0.0576	0.6085	0.4808	0.028*	
C18	0.02775 (7)	0.1177 (6)	0.39810 (18)	0.0222 (6)	
H18	0.0151	−0.0229	0.4078	0.027*	
C1	0.27747 (7)	0.1382 (6)	0.06967 (18)	0.0231 (6)	
H1A	0.2945	0.0338	0.0926	0.035*	
H1B	0.2859	0.3039	0.0669	0.035*	
H1C	0.2720	0.0801	0.0189	0.035*	

### Atomic displacement parameters (Å^2^)

**Table:**

	*U*^11^	*U*^22^	*U*^33^	*U*^12^	*U*^13^	*U*^23^
S1	0.0145 (3)	0.0139 (3)	0.0144 (3)	0.0006 (2)	−0.0002 (2)	0.0022 (2)
O1	0.0180 (9)	0.0144 (10)	0.0235 (10)	−0.0022 (8)	0.0015 (7)	0.0024 (8)
O2	0.0228 (10)	0.0240 (11)	0.0157 (10)	−0.0013 (8)	−0.0030 (7)	0.0036 (8)
O3	0.0277 (12)	0.0627 (18)	0.0240 (12)	0.0281 (13)	−0.0033 (9)	−0.0032 (12)
N1	0.0165 (11)	0.0146 (11)	0.0137 (10)	0.0022 (9)	−0.0020 (8)	−0.0027 (9)
C5	0.0145 (12)	0.0117 (12)	0.0152 (12)	0.0015 (10)	−0.0017 (9)	0.0012 (10)
C15	0.0172 (12)	0.0137 (13)	0.0154 (12)	0.0023 (10)	0.0018 (9)	0.0000 (10)
C6	0.0231 (13)	0.0142 (13)	0.0162 (12)	−0.0019 (11)	−0.0006 (10)	−0.0021 (10)
C21	0.0176 (13)	0.0172 (14)	0.0206 (14)	−0.0016 (11)	−0.0003 (10)	0.0011 (11)
C2	0.0156 (12)	0.0180 (14)	0.0174 (13)	0.0015 (10)	−0.0015 (9)	0.0038 (11)
C16	0.0131 (11)	0.0150 (13)	0.0169 (13)	0.0033 (10)	0.0014 (9)	0.0014 (10)
C10	0.0206 (13)	0.0148 (13)	0.0191 (13)	−0.0010 (11)	−0.0010 (10)	−0.0024 (11)
C22	0.0188 (13)	0.0355 (18)	0.0152 (13)	0.0079 (12)	0.0006 (10)	−0.0009 (12)
C17	0.0209 (13)	0.0151 (14)	0.0221 (14)	0.0009 (11)	0.0003 (10)	−0.0006 (11)
C14	0.0187 (13)	0.0177 (14)	0.0165 (13)	−0.0011 (11)	0.0005 (10)	−0.0034 (11)
C8	0.0151 (12)	0.0125 (12)	0.0123 (12)	0.0001 (10)	−0.0006 (9)	−0.0012 (10)
C9	0.0124 (11)	0.0144 (13)	0.0158 (12)	0.0021 (10)	−0.0009 (9)	−0.0016 (10)
C13	0.0189 (13)	0.0205 (14)	0.0206 (14)	−0.0010 (11)	0.0034 (10)	0.0003 (11)
C3	0.0268 (15)	0.0190 (15)	0.0251 (15)	−0.0022 (12)	0.0069 (12)	−0.0067 (12)
C4	0.0208 (14)	0.0169 (14)	0.0262 (15)	−0.0059 (11)	0.0034 (11)	−0.0064 (12)
C11	0.0285 (15)	0.0198 (15)	0.0180 (14)	0.0032 (12)	−0.0019 (11)	−0.0074 (11)
C19	0.0239 (14)	0.0286 (17)	0.0183 (13)	0.0051 (12)	0.0063 (11)	0.0065 (12)
C7	0.0195 (13)	0.0168 (14)	0.0213 (14)	−0.0057 (11)	−0.0016 (10)	0.0013 (11)
C12	0.0200 (13)	0.0288 (16)	0.0129 (12)	0.0047 (12)	0.0029 (10)	−0.0011 (11)
C20	0.0304 (16)	0.0231 (15)	0.0155 (13)	0.0014 (12)	0.0008 (11)	−0.0009 (12)
C18	0.0197 (14)	0.0179 (14)	0.0291 (15)	0.0008 (11)	0.0037 (11)	0.0065 (12)
C1	0.0191 (13)	0.0259 (16)	0.0245 (15)	0.0008 (12)	0.0032 (11)	0.0053 (13)

### Geometric parameters (Å, °)

**Table:**

S1—O2	1.432 (2)	C22—H22A	0.9700
S1—O1	1.441 (2)	C22—H22B	0.9700
S1—N1	1.612 (2)	C17—C18	1.395 (4)
S1—C5	1.762 (3)	C17—H17	0.9300
O3—C22	1.425 (4)	C14—C13	1.381 (4)
O3—H3A	0.8541	C14—C9	1.397 (4)
O3—H3B	0.8579	C14—H14	0.9300
N1—C8	1.476 (3)	C8—C9	1.506 (3)
N1—H1	0.8600	C8—H8	0.9800
C5—C4	1.389 (4)	C13—C12	1.393 (4)
C5—C6	1.394 (4)	C13—H13	0.9300
C15—C16	1.516 (4)	C3—C4	1.390 (4)
C15—C22	1.528 (4)	C3—H3	0.9300
C15—C8	1.552 (4)	C4—H4	0.9300
C15—H15	0.9800	C11—C12	1.387 (4)
C6—C7	1.388 (4)	C11—H11	0.9300
C6—H6	0.9300	C19—C20	1.384 (5)
C21—C20	1.389 (4)	C19—C18	1.389 (5)
C21—C16	1.390 (4)	C19—H19	0.9300
C21—H21	0.9300	C7—H7	0.9300
C2—C3	1.387 (4)	C12—H12	0.9300
C2—C7	1.392 (4)	C20—H20	0.9300
C2—C1	1.508 (4)	C18—H18	0.9300
C16—C17	1.397 (4)	C1—H1A	0.9600
C10—C11	1.389 (4)	C1—H1B	0.9600
C10—C9	1.397 (4)	C1—H1C	0.9600
C10—H10	0.9300		
			
O2—S1—O1	120.01 (13)	C13—C14—C9	121.2 (3)
O2—S1—N1	105.87 (13)	C13—C14—H14	119.4
O1—S1—N1	106.94 (12)	C9—C14—H14	119.4
O2—S1—C5	105.87 (12)	N1—C8—C9	113.2 (2)
O1—S1—C5	107.24 (12)	N1—C8—C15	105.4 (2)
N1—S1—C5	110.85 (12)	C9—C8—C15	114.1 (2)
C22—O3—H3A	123.7	N1—C8—H8	108.0
C22—O3—H3B	120.5	C9—C8—H8	108.0
H3A—O3—H3B	57.6	C15—C8—H8	108.0
C8—N1—S1	123.52 (19)	C14—C9—C10	118.3 (3)
C8—N1—H1	118.2	C14—C9—C8	120.8 (2)
S1—N1—H1	118.2	C10—C9—C8	120.9 (2)
C4—C5—C6	119.9 (3)	C14—C13—C12	120.0 (3)
C4—C5—S1	120.8 (2)	C14—C13—H13	120.0
C6—C5—S1	119.1 (2)	C12—C13—H13	120.0
C16—C15—C22	112.2 (2)	C2—C3—C4	121.5 (3)
C16—C15—C8	110.7 (2)	C2—C3—H3	119.2
C22—C15—C8	111.0 (2)	C4—C3—H3	119.2
C16—C15—H15	107.6	C5—C4—C3	119.5 (3)
C22—C15—H15	107.6	C5—C4—H4	120.2
C8—C15—H15	107.6	C3—C4—H4	120.2
C7—C6—C5	119.6 (3)	C12—C11—C10	120.3 (3)
C7—C6—H6	120.2	C12—C11—H11	119.8
C5—C6—H6	120.2	C10—C11—H11	119.8
C20—C21—C16	121.2 (3)	C20—C19—C18	119.9 (3)
C20—C21—H21	119.4	C20—C19—H19	120.1
C16—C21—H21	119.4	C18—C19—H19	120.1
C3—C2—C7	118.1 (3)	C6—C7—C2	121.3 (3)
C3—C2—C1	120.7 (3)	C6—C7—H7	119.3
C7—C2—C1	121.1 (3)	C2—C7—H7	119.3
C21—C16—C17	118.5 (3)	C11—C12—C13	119.5 (3)
C21—C16—C15	120.4 (3)	C11—C12—H12	120.2
C17—C16—C15	121.2 (3)	C13—C12—H12	120.2
C11—C10—C9	120.6 (3)	C19—C20—C21	119.9 (3)
C11—C10—H10	119.7	C19—C20—H20	120.0
C9—C10—H10	119.7	C21—C20—H20	120.0
O3—C22—C15	109.7 (2)	C19—C18—C17	120.0 (3)
O3—C22—H22A	109.7	C19—C18—H18	120.0
C15—C22—H22A	109.7	C17—C18—H18	120.0
O3—C22—H22B	109.7	C2—C1—H1A	109.5
C15—C22—H22B	109.7	C2—C1—H1B	109.5
H22A—C22—H22B	108.2	H1A—C1—H1B	109.5
C18—C17—C16	120.6 (3)	C2—C1—H1C	109.5
C18—C17—H17	119.7	H1A—C1—H1C	109.5
C16—C17—H17	119.7	H1B—C1—H1C	109.5

### Hydrogen-bond geometry (Å, °)

**Table:**

*D*—H···*A*	*D*—H	H···*A*	*D*···*A*	*D*—H···*A*
N1—H1···O1^i^	0.86	2.45	3.122 (3)	136.
O3—H3A···O3^ii^	0.85	2.06	2.910 (5)	179.7
C4—H4···O1	0.93	2.55	2.909 (4)	104.
C8—H8···O1	0.98	2.61	2.958 (3)	101.
C1—H1A···O2^iii^	0.96	2.63	3.557 (4)	161.
C1—H1B···O2^iv^	0.96	2.74	3.552 (4)	142.
O3—H3B···C16^ii^	0.86	2.67	3.489 (4)	160.1
O3—H3B···C17^ii^	0.86	2.85	3.499 (4)	133.7

Symmetry codes: (i) *x*, *y*+1, *z*; (ii) −*x*, *y*, −*z*+1/2; (iii) −*x*+1/2, *y*−1/2, −*z*+1/2; (iv) −*x*+1/2, *y*+1/2, −*z*+1/2.
